# VCNet: Hybrid Deep Learning Model for Detection and Classification of Lung Carcinoma Using Chest Radiographs

**DOI:** 10.3389/fpubh.2022.894920

**Published:** 2022-06-20

**Authors:** Ritu Tandon, Shweta Agrawal, Arthur Chang, Shahab S. Band

**Affiliations:** ^1^Institute of Advance Computing, SAGE University, Indore, India; ^2^Bachelor Program in Interdisciplinary Studies, National Yunlin University of Science and Technology, Douliu, Taiwan; ^3^Future Technology Research Center, College of Future, National Yunlin University of Science and Technology, Douliu, Taiwan

**Keywords:** capsule network, convolutional neural networks, CT, MobileNet, VCNet, VGG-16, Xception

## Abstract

Detection of malignant lung nodules from Computed Tomography (CT) images is a significant task for radiologists. But, it is time-consuming in nature. Despite numerous breakthroughs in studies on the application of deep learning models for the identification of lung cancer, researchers and doctors still face challenges when trying to deploy the model in clinical settings to achieve improved accuracy and sensitivity on huge datasets. In most situations, deep convolutional neural networks are used for detecting the region of the main nodule of the lung exclusive of considering the neighboring tissues of the nodule. Although the accuracy achieved through CNN is good enough but this models performance degrades when there are variations in image characteristics like: rotation, tiling, and other abnormal image orientations. CNN does not store relative spatial relationships among features in scanned images. As CT scans have high spatial resolution and are sensitive to misalignments during the scanning process, there is a requirement of a technique which helps in considering spatial information of image features also. In this paper, a hybrid model named VCNet is proposed by combining the features of VGG-16 and capsule network (CapsNet). VGG-16 model is used for object recognition and classification. CapsNet is used to address the shortcomings of convolutional neural networks for image rotation, tiling, and other abnormal image orientations. The performance of VCNeT is verified on the Lung Image Database Consortium (LIDC) image collection dataset. It achieves higher testing accuracy of 99.49% which is significantly better than MobileNet, Xception, and VGG-16 that has achieved an accuracy of 98, 97.97, and 96.95%, respectively. Therefore, the proposed hybrid VCNet framework can be used for the clinical purpose for nodule detection in lung carcinoma detection.

## Introduction

Cancer is still regarded as a dangerous disease with severe death rates. Lung cancer has the highest mortality or death rate of any cancer and is widely regarded as the deadliest carcinoma among all types of cancer. Consequently, several researchers are focusing on ways of detecting lung cancer nodules through digital images, specifically through computed tomography (CT). CT scans use X-rays to generate several images and create a challenge for radiologists to detect tiny nodules from these images (1). Analysis of nodules and their interpretation is the basic task performed by the radiologist for the diagnosis of lung cancer. Many scientists and researchers are working on automated solutions that will help doctors save time and money (2).

**Figure 1:**
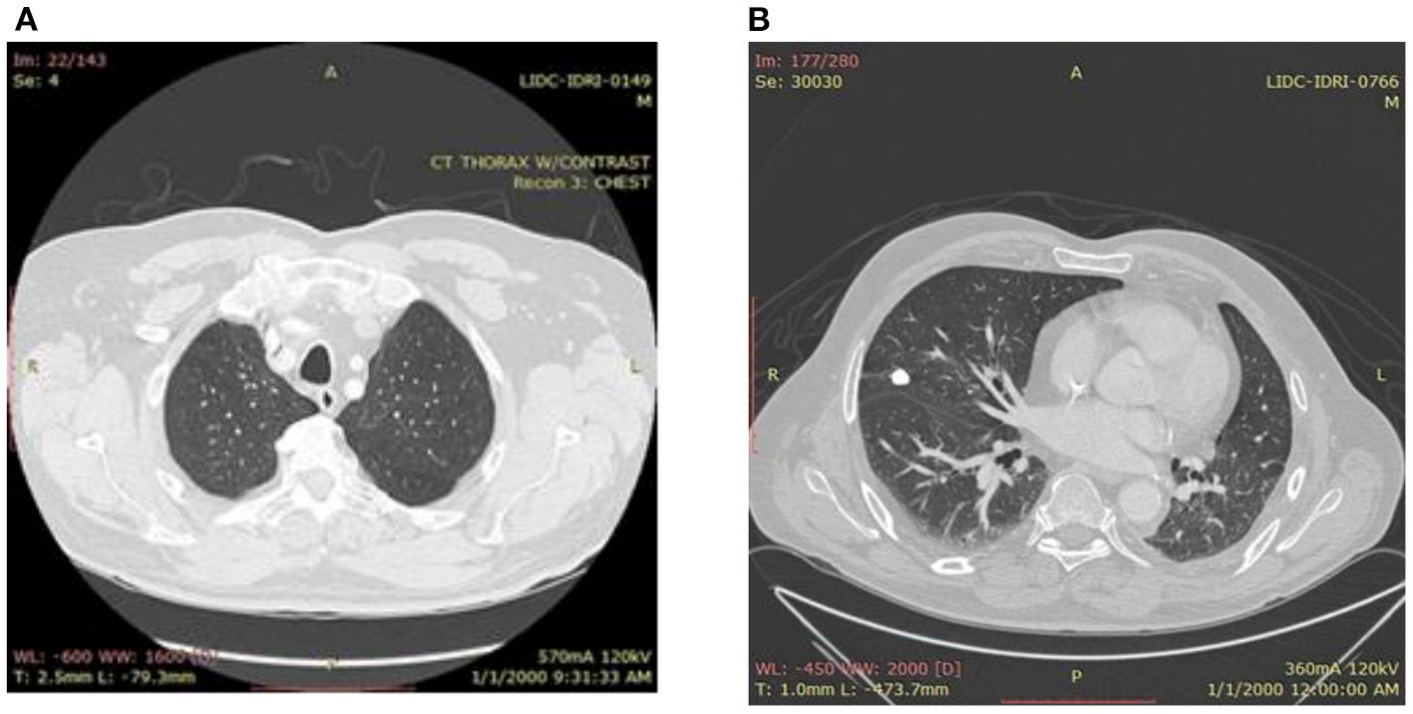
**(A)** Benign lung image. **(B)** Malignant lung.

In most cases, the size and appearance of nodules give the first inference regarding cancer and can be classified as benign or malignant. Generally, lung nodules <3 cm are considered normal nodules, and those larger than 3 cm are considered malignant or lung masses. Figures 1A,B shows the images of benign and malignant lung cancer. Based on nodule classification and other findings, cancer probability can be assessed. AI techniques are playing a very important role in the primary level detection and classification of various types of cancer (3). A variety of disciplines, including medicine, agriculture, games, and many more, have benefited from the deployment of deep learning (DL) models in recent years. In all of these fields, DL models perform quite well, particularly in specific types of applications such as classification of images, identification of objects, and image segmentation (4). DL is a subfield of artificial intelligence, having interconnected nodes to perform complicated tasks. Instead of using pre-programmed instructions, DL algorithms are capable of learning from the training data. Many of the researchers have already worked for lung carcinoma detection using deep learning (5, 6).

In computer vision and radiography, convolutional neural networks (CNN) are a class of artificial neural networks that are gaining a lot of popularity. CNN is used to automatically detect features from the images using a number of layers like convolution layers, pooling layers, and fully connected layers (7, 8). CNN uses successive convolution and pooling layers to classify the images. The pooling layer in the CNN reduces the dimension and classifies the object regardless of its spatial information. That means where the object is actually located in the image (9). This pooling function of CNN is both an advantage and a drawback. During the pooling function, it loses some important information that is very useful in image segmentation and object detection. To overcome this drawback, Geoffrey Hinton (10) proposed capsule network architecture.

A capsule is a cluster or group of neurons that stores information about a specific item in a picture; the information is mainly about its position, rotation, scale, etc. in a high-dimensional vector (8 dimensions or 16 dimensions), each. The capsule network architecture is divided into three parts. (1) primary capsule (convolution, reshape, and squash functions); (2) higher level capsule (dynamic routing); and (3) loss functions (margin and reconstruction loss).Capsule networks (CapsNet) (10) are basically used to overcome the loss of information that comes from the pooling operation of CNN and obtain spatial information. CapsNet's multiple convolutional layers are wrapped in capsules.

This paper utilizes transfer learning and presents a hybrid model, VCNet, for detecting the lung nodules from CT scans. Binary classification is done using the input of lung CT scans and the output is either “benign” or “malignant”. A lung CT image dataset is collected from LIDC-IDRI (11) and a new hybrid model that is a combination of VGG-16 and Capsule network is developed and applied for the classification and identification of lung cancer.VGG16 is one of the finest vision model architectures based on CNN computer vision techniques. The accuracy achieved by this model for ImageNet test data is 92.7%. The model presents an improved form of AlexNet and the main architectural changes are in filter size. The 16 in VGG is used to refer that it has 16 weight layers (12).

### Related Work

Lung cancer with the greatest death rate is the most prevalent and aggressive form in India. Patients with non-small cell lung cancer (NSCLC) have a 5-year survival rate of 18%. According to the 2018 GLOBOCON report (1), 11.6 percent of all lung cancer cases and 18.4 percent of all lung cancer deaths. Patients with lung cancer outnumber those with other cancers such as breast, liver, cervical, skin, and so on. Patients with pulmonary cancer can enhance their survival chances if they discover early lung cancer. Detection of cancer at last stage does not permit surgical treatment in most of the cases, so last stage lesions are treated by nonsurgical treatment like chemotherapy, Radiotherapy and immunotherapy. Computer vision methods proved an important result in lung nodule classification and detection at an early stage through CT scans. For converting the raw input into the desired features end to end learning allows direct mapping with elimination of hand –crafted features. Machine learning algorithms provide extremely good outcomes in computer vision technology and analysis of medical images. Two main classes, massive training artificial neural networks (MTANNs) and revolutionary neural networks may be performed with end-to-end training (CNNs).Nima Tajbakhsh et al. utilized both theoretical and practical approaches to compare these two classes of end-to-end learning for the detection of lung nodules and the differentiation of benign and malignant tumors in low-dose CT scans. For the analysis of the same the authors have used four MTANN architectures and two CNN architectures that all are having different depth. They used a substantial dataset for CNN training in the second phase, and their results demonstrate a smaller performance gap between MTANN and CNN. After both theoretical and experimental approach they had presented that MTANN gives better performance as compared to the CNN (13).

Goran Jakimovski et al. presented double CNN and regular CNN with a max pooling layer for identifying cancer stages through CT scans (14). One of the most difficult tasks in radiology imaging is automatically determining the precise position of the lung nodule. Hongtao Xie et al. presented the automatic detection of lung nodules using 2D CNN for the reading process of CT scans. The authors did their experiments on the LUNA-16 dataset and achieved a sensitivity of 86.42% (15). By combining information from PET and CT scans, Qin et al. (16) demonstrated a DL architecture for the noninvasive detection of lung cancer. The authors achieved a 0.92 area under the ROC curve. Nakrani et al. (17) presented a method for nodule detection using Resnet architecture in CT scans. The dataset used here is LIDC and the accuracy achieved is 95.24% (17).

Lu et al. (18) proposed an optimal methodology for detection of lung carcinoma at early stage. For good network accuracy & optimal arrangement Marine predators' algorithm is used.

Table 1 shows a summary of the research articles on lung cancer diagnosis and classification using the DL model CNN.

**Table 1:** Summary of the research articles on lung cancer diagnosis and classification using the DL model by CNN.

**Reference**	**Year**	**Purpose of study**	**Image acquisition**	**Image dataset**	**Accuracy/sensitivity**	**Major findings/limitations**
Jakimovski et al. (14)	2019	Lung cancer stage detection	CT images	LONI database	99.63%	A deep neural network has been designed to detect the stage of lung cancer. The pre-classification of photos is done using the K-mean approach
Xie et al. (15)	2019	Automated nodule detection	CT images	LUNA-16	86.42%	An automated pulmonary nodule detection-based model is proposed using two-dimensional CNN
Qi et al. (16)	2020	Classification of lung cancer	PET and CT images	Department of Radiology of the Henan Provincial People's Hospital	72% (Balanced Accuracy)	Used CT and PET multimodality noninvasive clinical images for lung cancer classification
Nakrani et al. (17)	2020	Lung nodule detection	CT images	LIDC-IDRI	95.24%	ResNet architecture of 2D convolutional neural network is used for detection of lung nodule
Neal Joshua et al. (19)	2021	Lung nodule detection	CT images	LUNA-16 dataset	97.17%	The 3D AlexNet architecture is being used. The 3D multi-dimensional convolutional neural network is used for lung nodule detection
Bharati et al. (20)	2020	Lung disease detection	X-ray images	Chest X-ray dataset of NIH collected from kaggle	73%	Proposed a hybrid model for lung disease detection using a modified capsule network
Afshar et al. (21)	2020	Lung nodule malignancy prediction	CT images	LIDC-IDRI	83%	The 3D multi-dimensional convolutional neural network is used for lung nodule detection

### Problem Statement

Many researchers have already presented various DL models for lung cancer detection, but the required accuracy on public datasets is still a big challenge for using those models for clinical purposes and also for radiologists to diagnose the cancerous nodules. Some of the researchers have achieved good accuracy in their work, but they have used either private datasets collected from hospitals or some small datasets available on the internet. In Tajbakhsh et al. (13), 95% accuracy was attained by comparing two end-to-end classes that are large artificial neural network training and convolutional neural networks. But for the implementation of the same, they used the unpublished dataset of 31 patients, including 38 scans collected from the participation of a lung cancer screening program. In (19), the LUNA-16 dataset was used to detect the lung nodules using the 3D AlexNet architecture of CNN and achieved an accuracy of 97.17%. In Jakimovski and Davcev (14), the achieved accuracy was 99.63% for lung cancer stage detection, but the authors of this paper used the private dataset of the University of South California.

To the best of our knowledge, the paper (20) implemented a hybrid model using a modified capsule network for lung disease detection and achieved an accuracy of 73%. For the detection of lung nodules, a multi-scale capsule network is proposed in (21) and achieved an accuracy of 83% for the LIDC dataset.

Based on these works the major contributions of our work are:

- A hybrid model named as VCNet is proposed by combining the features of VGG-16 and capsule network (CapsNet).
- A pre-trained VGG-16 model is used for feature extraction.
- CapsNet layers are used to handle misalignment problems with the existing deep learning models.
- Fully connected layers along with dropouts and sigmoid activation functions are also used to prevent overfitting and to build a generalized model.
- The performance of VCNet is verified on the Lung Image Database Consortium (LIDC) image collection dataset.

## Materials and Methods

The current investigation was performed to determine if lung CT images were benign or malignant.

**Figure 2:**
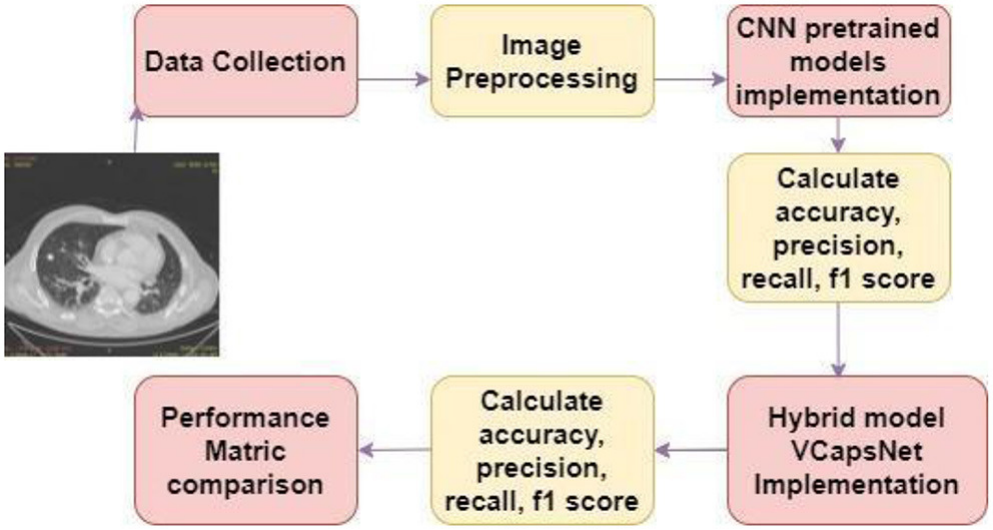
Methodology.

Figure 2 shows a diagrammatic depiction of the proposed seven-stage technique. Stage I includes the collection of CT images of lungs from LIDC IDRI dataset. Stage-II includes image pre-processing. Stages III and IV consist of the study & implementation of pre-trained CNN models like VGG, Xception and MobileNet and calculate their performance by accuracy, precision, F1 score and recall. Study, implementation, and performance analysis of hybrid model VCNet is shown in stages V & VI. Stage VII is the last phase, VCNet classifies CT scans as benign or malignant and analyzes their performance in terms of accuracy, precision, F1 score, and recall. All these stages are described in subsections.

### Dataset Description

Dataset used here is LIDC- IDRI (11) that has a total 1,018 scans of 1,010 patients with 244,527 images. Each case in this dataset holds images of the CT scan and their corresponding XML file that contains annotations of the CT scan. Four experienced thoracic radiologists performed these annotations in two phases. In the first step, each radiologist individually categorized CT results into three classifications (nodules that are ≥3 mm, nodules that are ≤ 3 mm and non-nodule that are ≥3 mm). After that in the second step, classification is reviewed by each radiologist along with classification by another radiologist secretly. So every nodule of the dataset is assessed by all four radiologists separately. In this dataset, the diagnosis is done at two label patient levels and the nodule level. The DICOM images of the CT scan vary from 64 to 764 slices and have a resolution of 512^*^512^*^width. The average width of CT scans in this dataset is 240. Lung nodules in the LIDC-IDRI dataset are classified into four classes. (1) Unknown (No Label), (2) Benign or normal or non-cancerous (3) Primary lung cancer or Malignant, (4) Metastatic Lesion whose primary cancer was other than lung cancer.

### Data Pre-processing

Preprocessing of the images is required to minimize the network overhead and computational complexity. The LIDC-IDRI dataset consists of various lung CT scan images and labeling is provided into csv file. For implementing the proposed model segregation and labeling of cancerous and noncancerous images are done based on annotations presented in csv file. Collected Dicom images are converted into jpg files using Radiant Dicom viewer 64 bit. Data augmentation methods used are rescaling, rotation, horizontal and vertical flip, and utilized ImageDataGenerator library of Keras for implementation. Rescaling is used to limit the image pixels between 0 to 1 and it helped in reducing computational complexity. Color Transformation of images (Gray to RGB) is done for better visualization. Rotation is done in a range of 10 means images are rotated from 0 to 10 degrees.

We have taken rotation range= 1 degree to randomly rotate our image between 0 and 1 degree. Rescale 1. /255 is done to transform every pixel value from range [0, 255] → [0, 1]. Color mode function of flowfrom directory of python is used to convert gray images into RGB images. For resizing the image from 512^*^512 to CNN input size, Keras ImageDataGenerator class is used that provides a quick and easy way to augment images.

The flow_from_directory () method of ImageDataGenerator is used to read the images directly from the directory and augment them while the neural network model is learning on the training data. The main important parameter of this method is target_size: Size of the input image. It is an attribute which converts images into size provided for the input. In our case we have taken image size 512^*^512, to set traget_size attribute value.

### Convolutional Neural Network

Convolutional, pooling, and fully connected layers make up the three main layers of CNN design. For CNN classification and pattern recognition, the most popular architectures are LeNet, AlexNet, VGG-Net, Res-NET, ZF-Net, and SqueezNet. Due to CNN's simplicity in calculation and function, the backpropagation algorithm is one of the finest learning algorithms. Back propagation differentiates during training between the algorithm predicting of labels and the ground truth labels computed by the loss function. Equation 1 gives the form of cross-entropy. Training samples are represented as inputs, and the true values of output neurons (j) are represented as inputs in the output layer ($a_{j}^{l}$) in this equation; *n* is the total number of training samples. It can be computed as:


(1) $$\begin{array}{l} C=-\frac{1}{n}\sum \;\times \;\sum_{j}\left(y_{j\;}\;a_{j}^{l}\;\right)\;\left(1+y_{j\;}\right)ln\left(1-a_{j}^{l\;}\right). \end{array}$$

### Convolutional Layer

Each feature map comprises groups of neurons that together make up a feature map. Convolutional layer output is calculated by Eq. 2. Kernel (filter) size, feature maps, bias, and weight of a kernel are represented by *F, m, B*, and *W*_*j*_, respectively. Convolutional layers have an output known as *y*_i_, where *I* is an index that denotes the *i*th feature map in a layer known as *l* (2).


(2) $$\begin{array}{l} y_{i}^{l\;}=B_{i}^{l}+\;\overset{ml\left(l-1\right)}{\underset{j=1}{\sum }}F_{i,j}l\;\times W_{j}^{l-1} \end{array}$$

### Pooling Layer

**Figure 3:**
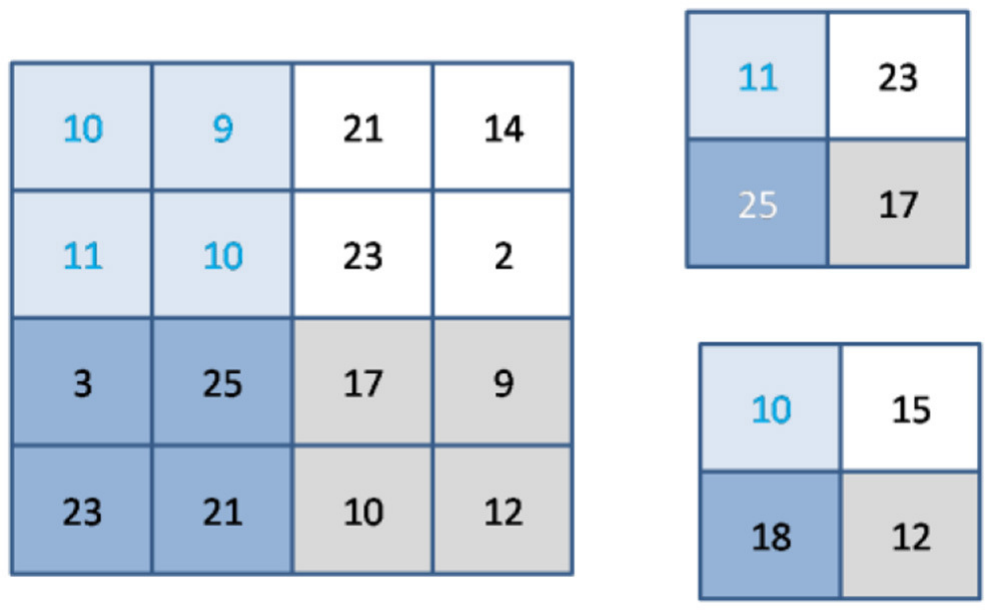
Example of max & average pooling.

Generally, pooling layers are employed in collaboration with convolutional layers; as a result, sub-sampling decreases the input size in all depth portions and thus helps prevent over-fitting while training the network. The pooling process reduces the amount of the input, and as a result, the depth dimension is not altered. Maximum and average pooling is the most commonly used pooling technique, as shown in Figure 3.

There are two Equations in (3) that determine the breadth and height of the pooled-layer output.


(3) $$\begin{array}{l} \;B_{2}=\;\left(\frac{B_{1}+\;F}{S}\right)+1 \end{array}$$

(4) $$\begin{array}{l} \;H_{2}=\;\left(\frac{H_{1}+\;F}{S}\right)+1 \end{array}$$

### Relu Activation

**Figure 4:**
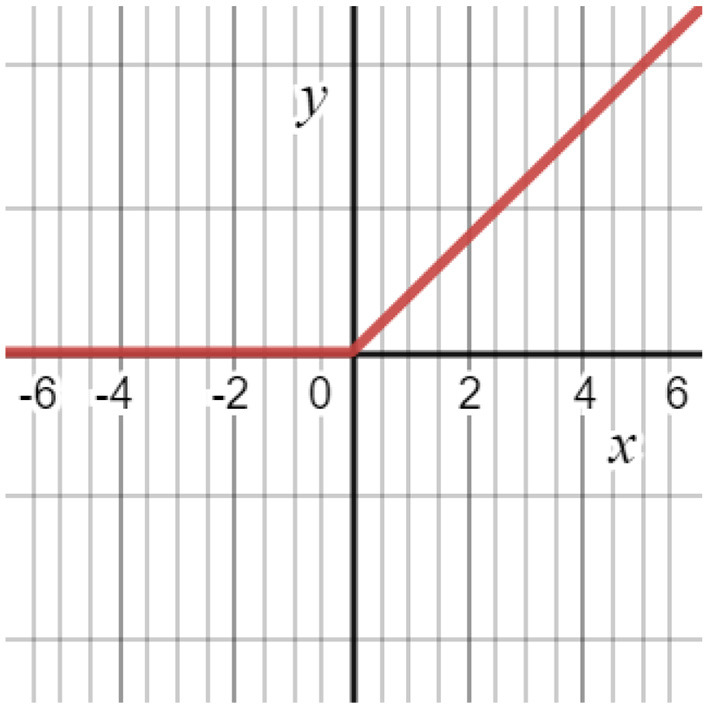
ReLu activation function.

A nonlinear activation function is used in CNN instead of a local connection to determine the output of neurons. Using the improved performance, faster learning, and simple structure as a benefit, it is advantageous to use the logistic sigmoid and hyperbolic tangent activation functions (Figure 4). Equation (5) illustrates the Relu function. The gradient of the Relu function is zero if y is less than zero; otherwise, it is one (2).


(5) $$\begin{array}{l} f\left(y\right)=max\left(0,x\right) \end{array}$$

### Softmax Activation

Softmax is used to determine the probability of each ground truth label producing an output value between 0 and 1, then the results are transformed to perceptible values. The softmax function's formula is given by equation (6). *f* (*z*) (22) transforms random variables (*z*) into meaningful values between 0 and 1 by using the softmax function.


(6) $$\begin{array}{l} \begin{array}{c} f{\left(z\right)}_{i}=\frac{e^{z_{j}}}{\sum_{k=1}^{k}e^{z_{k}}}\;for\;j=1. \end{array} \end{array}$$

### Performance Metrics

To examine the developed hybrid DL model for lung cancer nodule identification and classification using various performance metrics like accuracy, precision, recall, F1-score, and support, Overall accuracy is calculated as the total true results divided by the total number of samples. The blocks are derived from the pulmonary pictures in this study. These metrics are specified in several words like “True Positive” (TP) or “False Negative” (TN) and “False Negative” (FN). These are defined by different terminologies. TP is computed in this context as the suspected lung nodules that have been diagnosed properly as malignant. TN is determined by the number of pictures that are categorized as benign. The FN term is computed by the method that does not detect cancerous nodules. In addition, FP is determined by calculating the number of pictures not properly identified for lung cancer. Sensitivity and specificity are terms used to describe the true positive and true negative rates (23).The formulas for calculating these performance parameters are presented through Eqs 1–4.


(7) $$\begin{array}{l} Accuracy=\;\frac{TP+TN}{TP+TN+FP+FN} \end{array}$$

(8) $$\begin{array}{l} Recall=\;\frac{TP}{TP+FN} \end{array}$$

(9) $$\begin{array}{l} Precision=\frac{TP}{TP+FP} \end{array}$$

(10) $$\begin{array}{l} F1\;Score=2\;\times \frac{Precision\;\times recall}{Precision+recall}. \end{array}$$

### Experimental Setup

The experiments are performed on CNN Pretrained models which are VGG-16, MobileNet and Xception on LIDC dataset. The new model VCNet is also developed and used for experimental purposes. The VCNet is designed by using a hybrid combination of VGG and Capsule Network.

### Pretrained Models

We have used the following pre-trained models for the detection and classification of lung cancer using the LIDC-IDRI dataset.

#### Mobilenet

MobileNet employs depth-wise separable convolutions, which means that instead of combining all three and flattening, it executes a separate convolution on each color channel. The input channels are filtered as a result of this. For MobileNets, depth wise convolution applies a single filter to each input channel. The depth wise convolution's outputs are then combined using an 11 convolution by the point wise convolution. In one step, a conventional convolution filters and combines inputs to create a new set of outputs. The depth wise separable convolution divides this into two layers: one for filtering and the other for combining. This factorization results in a significant reduction in computation and model size (23).

#### Xception

Xception is a Depth wise Separable Convolutions-based deep convolutional neural network architecture. Google researchers came up with the idea. Inception modules in convolutional neural networks are described by Google as an intermediate step between normal convolution and the depth wise separable convolution operation (a depth wise convolution followed by a point wise convolution). In this sense, a depth wise separable convolution can be thought of as an Inception module with the most towers possible. This result leads them to propose new deep convolutional neural network architecture based on Inception, but with depth wise separable convolutions in place of Inception modules (24).

#### VGG-16

VGG16 as a convolutional neural network architecture in their study “Very Deep Convolutional Networks for Large Scale Image Recognition.” On Image Net, a dataset of over 14 million images belonging to thousands of classifications, this model obtains 92.7 percent accuracy. It employs multiple 3 × 3 filters, one after the other. VGG16 was trained on the ImageNet dataset over weeks using the Nvidia Titan Black GPU. There are 13 convolution layers, five max-pooling layers, and three dense layers in the VGG16 pre-trained architecture. Changes to the VGG-16 algorithm include a global average pooling layer and two dense layers with the ReLu and Softmax activation functions. Both dense layers have a dropout rate of 0.5 (13).

#### Resnet

ResNet introduced by Kaiming He in 2015, it has become key work proving that incredibly deep networks may be trained using regular SGD with additional accuracy by changing the residual module to incorporate identity mapping, as proven in this study. This network employs a VGG-19-inspired 34-layer plain network architecture, after which the shortcut connection is implemented. The design is subsequently transformed into a residual network as a result of these shortcut connections (25).

#### Inception V3

Inception-v3 is a convolutional neural network design from the Inception family that includes Label Smoothing, Factorized 7 × 7 convolutions, and the inclusion of an auxiliary classifer to transport label information deeper down the network, among other improvements. Inception v3 is a commonly used image recognition model that has been demonstrated to achieve an accuracy of better than 78.1 percent on the ImageNet dataset. The model represents the sum of several concepts produced over the years by numerous scholars (26).

Table 2 presents the architecture details and number of parameters used for MobileNet, Xception, and VGG-16, ResNet & Inception V3.

**Table 2:** Model architecture.

**S.No**.	**Model**	**Size**	**TOP 1 accuracy**	**TOP 5 accuracy**	**No of parameters**
1	MobileNet	17 MB	0.665	0.871	4,253,864
2	Xception	88 MB	0.790	0.945	22,910,480
3	VGG-16	528 MB	0.715	0.901	138,357,544
4	ResNet	99 MB	0.759	0.929	25,636,712
5	Inception V3	92 MB	0.788	0.944	23,851,784

### VCNeT Model Building and Implementation

The proposed hybrid model is implemented on the Google Colab platform by utilizing the python libraries Tensorflow and Keras. For the transfer learning pre-trained model of CNN, VGG-16 is used and modified by adding global average pooling layers and dense layers with ReLu activation function. After the VGG, a capsule network has been added with route 03. Our results show that the hybrid model VCNet performs better than the VGG model, Xception, and MobileNet architectures.

**Figure 5:**
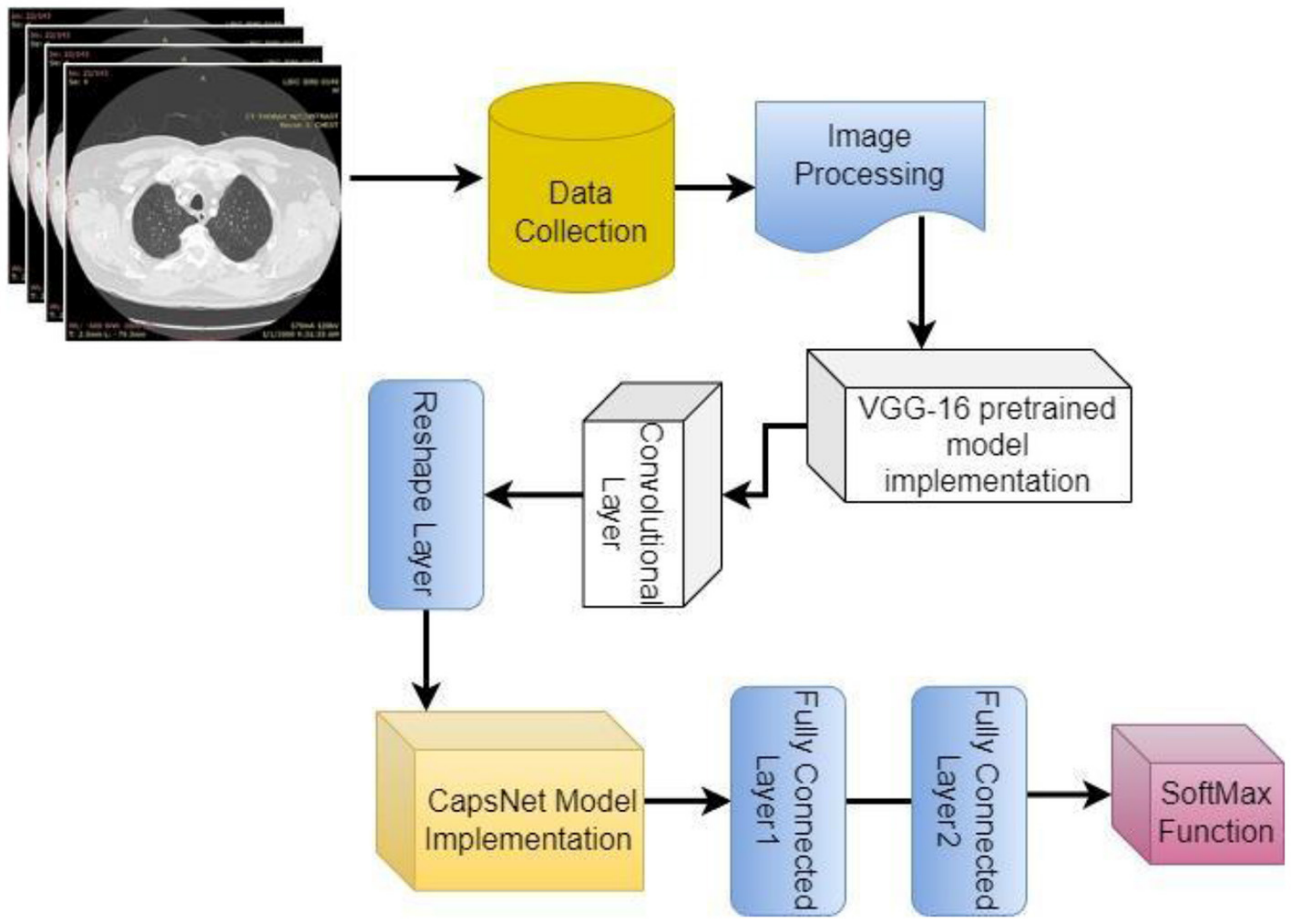
Architecture of VCNet.

**Figure 6:**
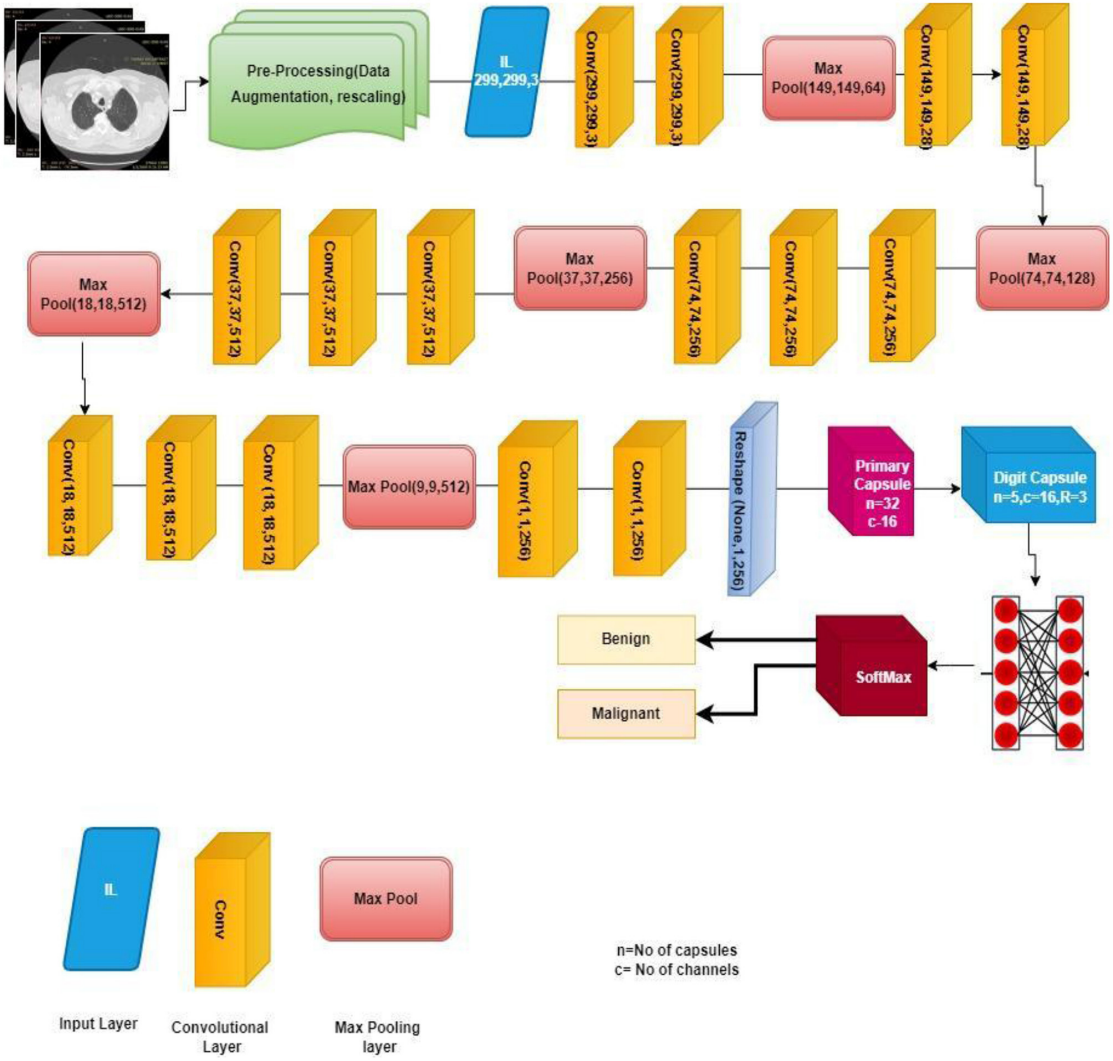
Proposed VCNet for the classification of lung nodule.

Figure 5 shows the complete architecture of the VCNet and summary of the proposed model is shown in Figure 6.

### Implementation of Capsule Network

**Figure 7:**
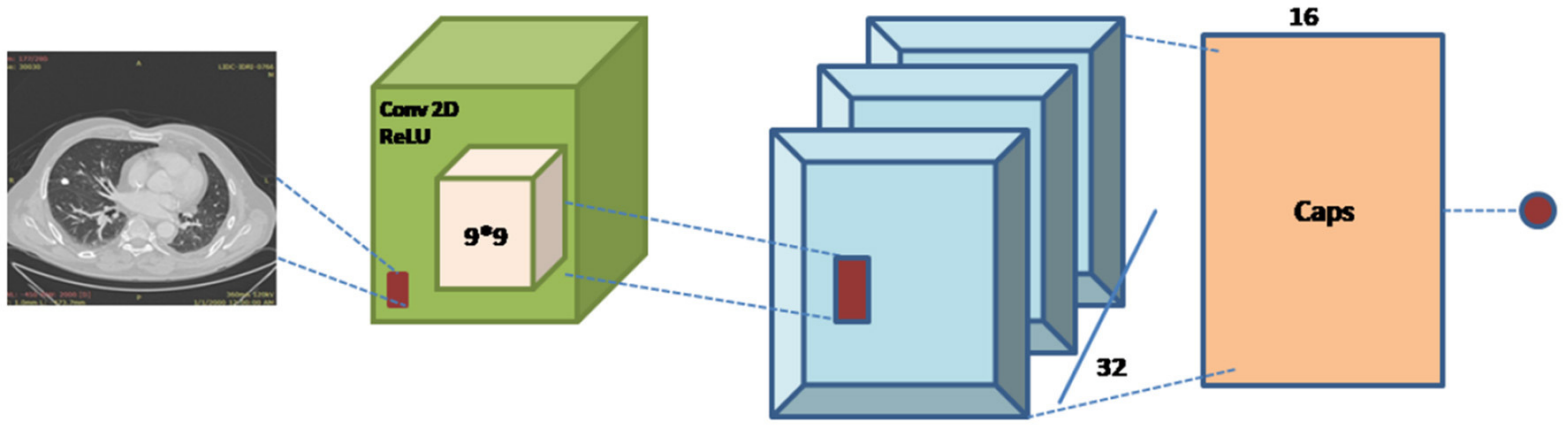
CapsNet architecture.

For the implementation of capsule network Global_Average_Pooling layer is used as an input and after that Convolution 2-dimensional layer is used to extract features with a kernel size of 9,9,512 and these features are mapped using reshape function to convert the images into one-dimensional array using reshape (1,1,3) along with squash function which is used for normalization. Lambda ?? is used after this to show that the lung image characteristics had a normal value of 0 with the default route transfer rate [−0.5: 0.5]. Calculating a margin and reconstruction loss is the most recent loss computation. This article uses the CapsNet for lung imaging data from Hinton's major architecture (26). Figure 7 shows a basic architecture of capsule network (CapsNet) for analysis of lung nodules.

The capsule network architecture is summarized as follows:

The first Convolution layer with kernel_size = 9, filters = 512, padding = “same,” strides = 9, activation = “relu,” dim_capsule = 8, *n*_channels = 32, padding = “valid,” strides = 2, kernel_size = 9 for the second primary capsule. *n*_class = num_capsule, dim_capsule = 16, stable of the set routings for the diagnosis capsule.

## Result

**Figure 8:**
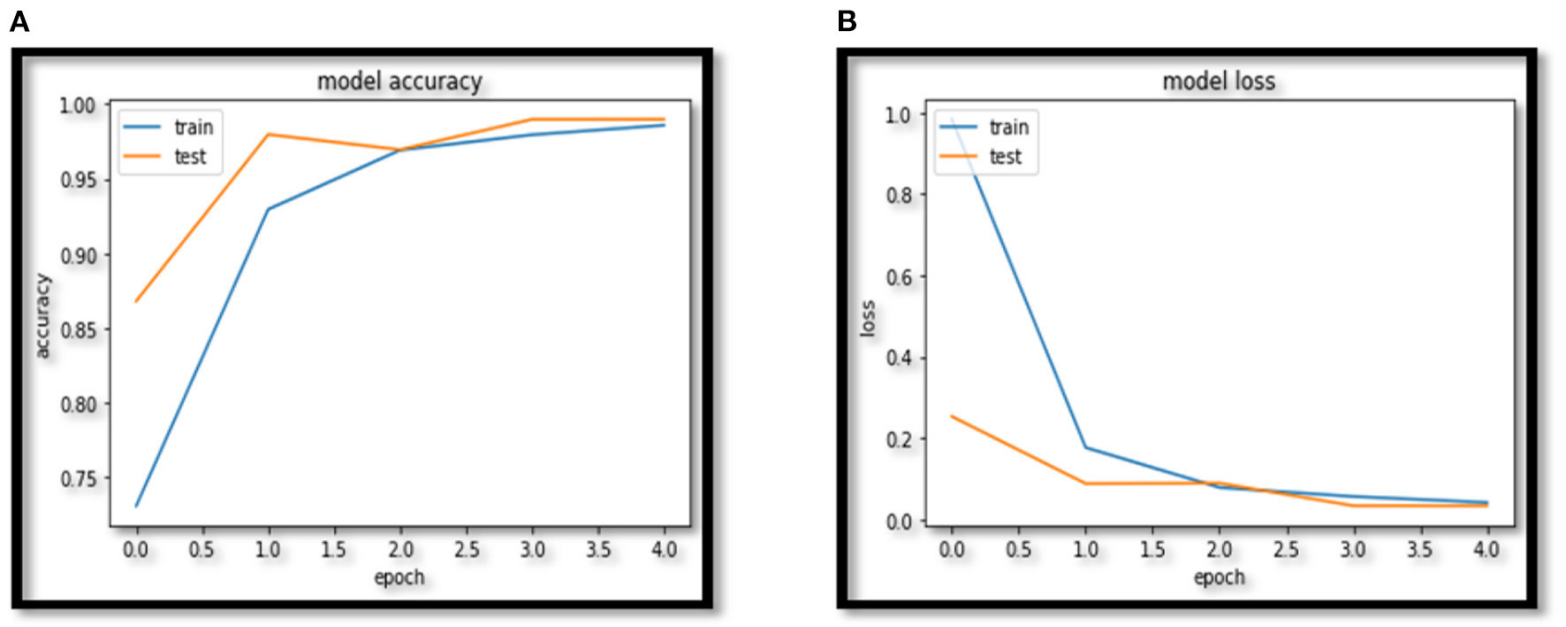
**(A)** MobileNet accuracy. **(B)** MobileNet loss.

**Figure 9:**
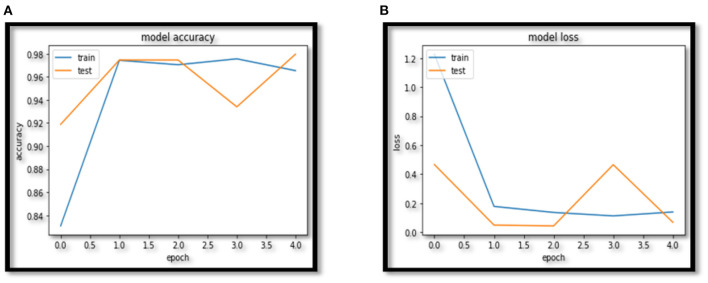
**(A)** Xception accuracy. **(B)** Xception loss.

**Figure 10:**
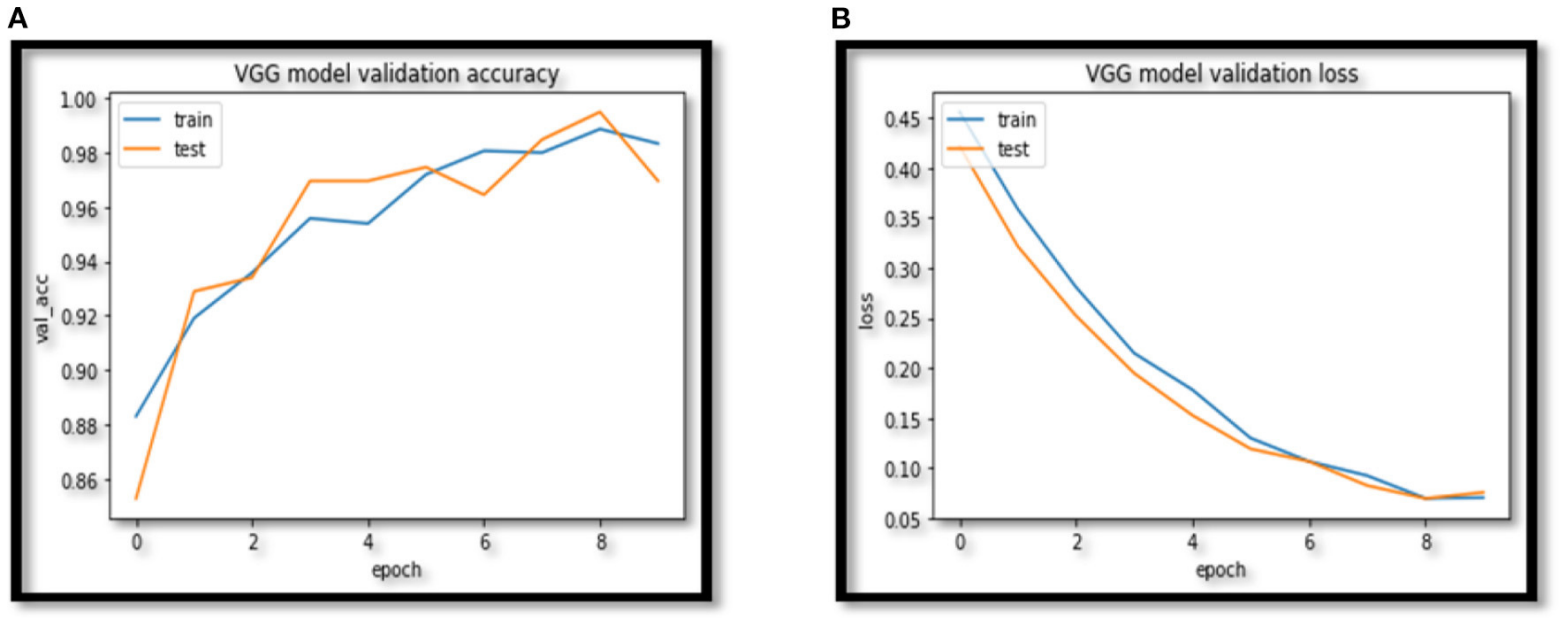
**(A)** VGG accuracy. **(B)** VGG model loss.

**Figure 11:**
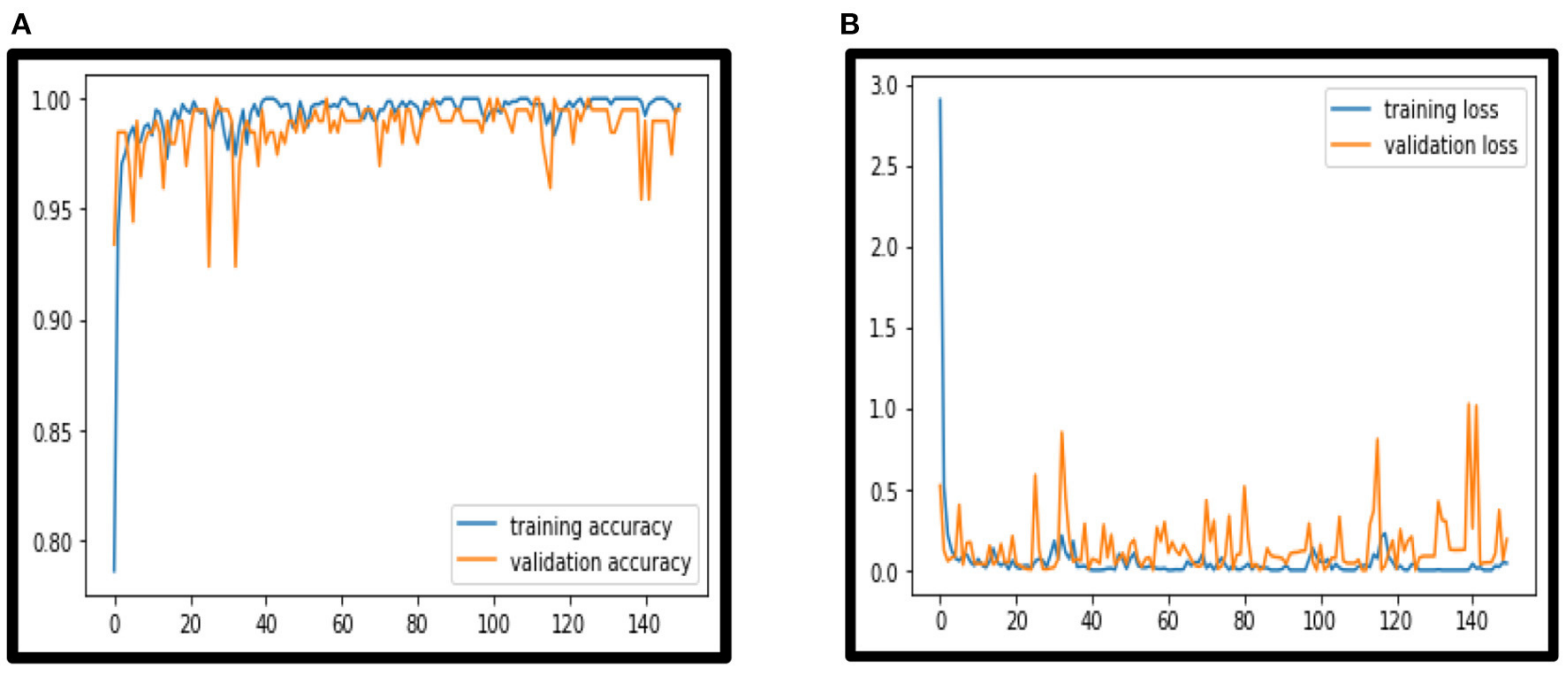
**(A)** VCNet accuracy. **(B)** VCNet loss.

In this paper VCNet, a new hybrid DL model is proposed for the detection of lung carcinoma using CT images. The proposed model is applied to CT images collected from the LIDC-IDRI dataset. The accuracy presented by VCNet architecture is 99.49% which is the highest among other used pretrained models. The pretrained models MobileNet, Xception, and VGG-16 achieved accuracy of 98, 97.97 and 96.95% respectively. The hybrid model built is evaluated with a validation set of pulmonary pictures. The accuracy and loss graphs of MobileNet architecture are illustrated in Figures 8A,B, 9A,B, Xception architecture accuracy and loss graph, Figures 10A,B, VGG-16 architecture accuracy and loss graph and Figures 11A,B represent the accuracy and loss graph for VCNet's proposed architecture.

The findings in Table 3 shows that suggested hybrid model VCNet were more accurate than other models in classifying lung CT images. As a consequence, the suggested hybrid VCNet attained the highest accuracy, which was 99.49 percent.

**Table 3:** Comparative analyses in terms of accuracy among the proposed VCNET and the State-of-the-Art models.

**Reference**	**Year**	**Image dataset**	**Accuracy**
Jakimovski et al. (14)	2019	LONI Database	99.63%
Xie et al. (15)	2019	LUNA-16	86.42%
Qi et al. (16)	2020	Department of Radiology of the Henan Provincial People's Hospital	72%
Nakrani et al. (17)	2020	LIDC-IDRI	95.24%
Neal Joshua et al. (19)	2021	LUNA-16 dataset	97.17%
Afshar et al. (20)	2020	Chest X-ray dataset of NIH collected from kaggle	73%
Bharati et al. (21)	2020	LIDC-IDRI	83%
**Proposed VCNet**	**2022**	**LIDC-IDRI**	**99.49%**

Classification matrix of different models for detection of lung nodules using LIDC IDRI dataset is presented in Table 4.

**Table 4:** Classification matrix of different models using LIDC dataset.

**Model**	**Accuracy**	**F-Measure**	**Sensitivity**	**Specificity**	**AUC**
CNN	95.88 ± 2.01	95 ± 1.84	96 ± 1.85	96 ± 1.92	96 ± 1.02
Xception	97.97 ± 1.02	96.87 ± 1.82	97.47 ± 1.05	97.97 ± 1.02	96.97 ± 1.02
VGG-16	96.95 ± 1.75	96.95 ± 1.55	95.95 ± 1.75	96.95 ± 1.25	95.95 ± 1.84
MobileNet	98 ± 0.40	98 ± 0.55	98 ± 0.65	98 ± 0.40	98 ± 0.35
ResNet	93.5 ± 1.38	93.5 ± 1.55	93.5 ± 1.28	93.5 ± 1.4	93.5 ± 1.38
Inception V3	94.35 ± 1.1	94.35 ± 1.15	94.35 ± 1.2	94.35 ± 1.10	94.35 ± 1.1
VCNet	**99.49** **±** **0.2**	**99.12** **±** **0.5**	**99.25** **±** **0.5**	**99.25** **±** **0.6**	**99.12** **±** **0.6**

**Figure 12:**
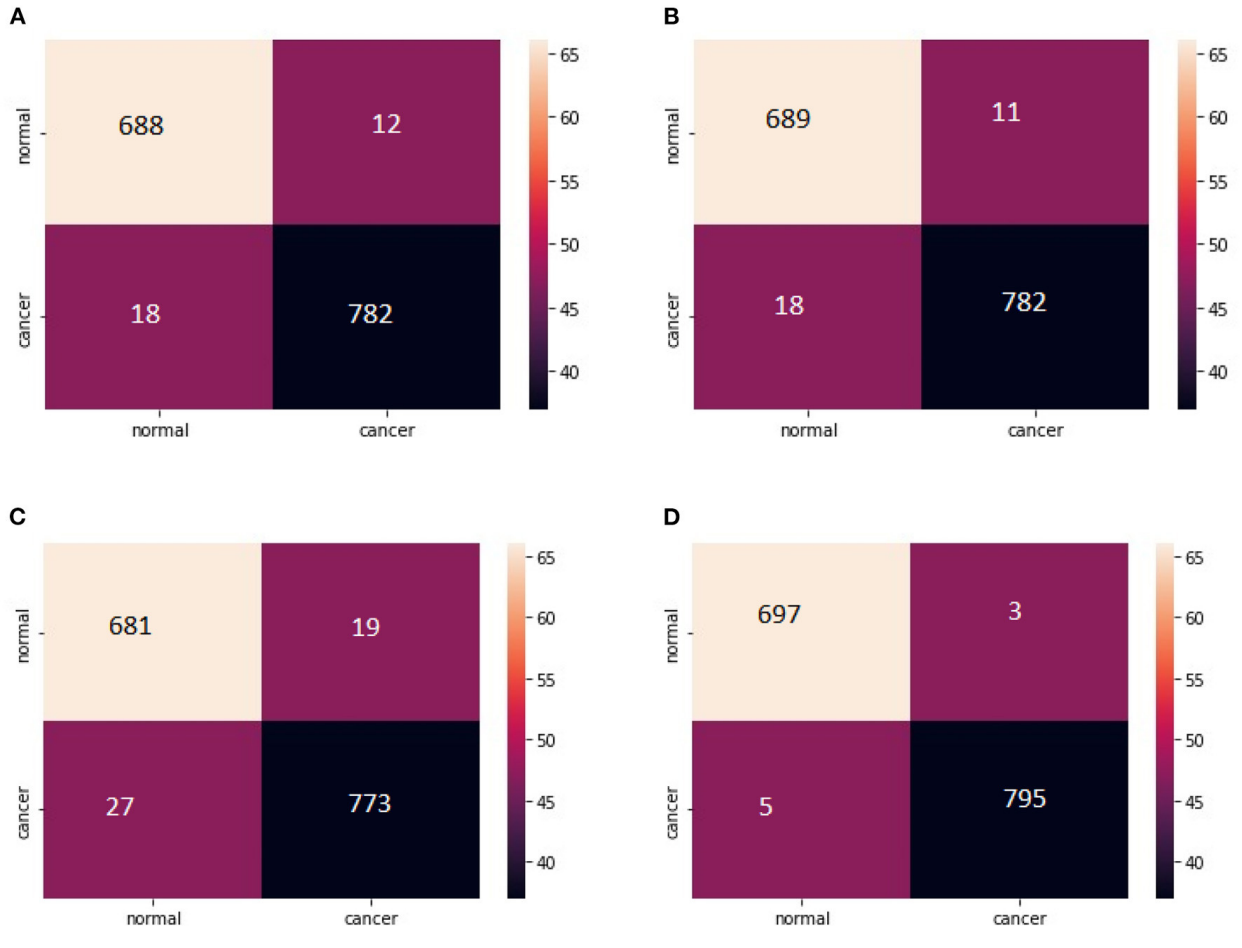
**(A)** Confusion Matrix MobileNet. **(B)** Confusion Matrix XceptionNet. **(C)** Confusion matrix VGG-16. **(D)** Confusion matrix VCNet.

The total number of properly identified lung nodules and their related erroneous predictions are shown in the confusion matrix created after evaluation in Figures 12A–D. For the statistical analysis the performance of the hybrid model VCNet is compared with various competitive models such as CNN, MobileNet, Xception, VGG-16, ResNet and InceptionV3.

## Discussion

There are numerous DL models for lung cancer diagnosis; nevertheless, accuracy in public datasets is still a significant obstacle (27). Overall, the researchers' results were accurate, although some of them utilized either private hospital datasets or tiny datasets that were publicly available on the internet. 95% accuracy was obtained by comparing two big artificial neural network end-to-end classes, one of which was the last stage of convolutional neural network training and the other the final stage of artificial neural network training. The implementation of the same employed the unpublished dataset of 31 patients that had 38 scans (13).

Detecting malignant lung nodules from the CT images takes a significant amount of time, especially if you don't have prior training in the subject. Despite recent progress in using deep learning models for lung carcinoma detection, clinical use is still difficult. For the majority of the cases, deep learning neural network models are used to identify the main nodule of the lung while ignoring the tissue that surrounds it.

In this section, the output of VCNet architecture and pre-trained models of CNN VGG-16, MobileNet and Xception on collected lung CT images are presented for classification of lung cancer nodules as normal and malignant. There were no issues applying the models because of the LIDC-IDRI dataset after preprocessing.

According to this study, 80 % of said dataset is used as a training set and the remaining 20 percent is used to evaluate the suggested VCNet architectures and pre-trained VGG-16. MobileNet& Xception architectures. Table 4 provides a summary of the Accuracy, Precision, Recall, and F1 scores for each of the four architectures.

The model's validation accuracy and loss curves are shown in Figures 8–11. Using the graphs, we may conclude that no over fitting or under fitting has occurred. Training and validation accuracy and losses continue to converge, and they reach a maximum at the end of 30 epochs. On the proposed VCNet, examined in terms of validation accuracy, it was determined that the network has an error rate of <0.1% and a test accuracy of <0.1% ,demonstrating a good correlation between the actual and predicted values.

For lung nodules, the estimated values of Specificity and Sensitivity are near to 1 shown in confusion matrix, indicating that the model has a high true positive and true negative rate. Furthermore, the low false positive and negative rates indicate that the proposed VCNet model has a very low risk of misclassifying data. As a result, the algorithm was able to properly detect lung nodules with extremely little error.

The proposed hybrid model evaluated on the Lung Picture Database Consortium image collection dataset achieved higher accuracy as compared to current techniques MobileNet, Xception, and VGG-16. It is critical for any system in the medical sector to produce accurate findings with a little mistake, since this framework allows for clinical purposes; it can be utilized for the detection of lung nodules in lung carcinoma.

## Conclusion

As a result of deep learning methods' impressive outcomes, in this research, CNN-based hybrid architecture, one of the effective models of deep learning in medical research, was applied to examine lung cancer on CT scan pictures of the LIDC-IDRI dataset. Therefore, prior to implementing the model, a collection of CT scan pictures was generated from an image dataset. The proposed hybrid model is applied to CT images collected from the LIDC-IDRI dataset. The proposed VCNet architecture proposed a higher accuracy of 99.49% while the existing methods of MobileNet, Xception, and VGG-16 achieved an accuracy of 98, 97.97, and 96.95%, respectively. This proposed hybrid VCNet framework can be used for clinical purposes for nodule detection in lung carcinoma detection, which will be a very useful and time-saving method for radiologists.

### Future Scope

In the future this hybrid model can be used for the detection of other types of cancer like Breast, Liver, Cervical, Brain, and Skin etc. The performance of the model can be optimized by using various optimization techniques also.

## Data Availability Statement

The original contributions presented in the study are included in the article/supplementary material, further inquiries can be directed to the corresponding authors.

## Author Contributions

RT: review of literature, methodology, experiments and result preparation, and draft preparation of paper. SA: methodology, proof reading of paper, and experimental design. AC: review of literature, result preparation and funding support. SB: proof reading of paper and funding support. All authors contributed to the article and approved the submitted version.

## Conflict of Interest

The authors declare that the research was conducted in the absence of any commercial or financial relationships that could be construed as a potential conflict of interest.

## Publisher's Note

All claims expressed in this article are solely those of the authors and do not necessarily represent those of their affiliated organizations, or those of the publisher, the editors and the reviewers. Any product that may be evaluated in this article, or claim that may be made by its manufacturer, is not guaranteed or endorsed by the publisher.
